# Endpoints for geroscience clinical trials: health outcomes, biomarkers, and biologic age

**DOI:** 10.1007/s11357-022-00671-8

**Published:** 2022-10-19

**Authors:** Steven R. Cummings, Stephen B. Kritchevsky

**Affiliations:** 1 San Francisco Coordinating Center, California Pacific Medical Center Research Institute, San Francisco, CA USA; 2 Departments of Epidemiology and Biostatistics, University of California, San Francisco, CA USA; 3 Wake Forest University School of Medicine, Sticht Center for Health Aging and Alzheimer’s Prevention, Wake Forest, NC USA

**Keywords:** Geroscience, Biological age, Epigenetic age, Surrogate marker, Clinical trials

## Abstract

Treatments that target fundamental processes of aging are expected to delay several aging-related conditions simultaneously. Testing the efficacy of these treatments for potential anti-aging benefits will require clinical trials with endpoints that reflect the potential benefits of slowing processes of aging. There are several potential types of endpoints to capture the benefits of slowing a process of aging, and a consensus is needed to standardize and compare the results of these trials and to guide the analysis of observational data to support trial planning. Using biomarkers instead of clinical outcomes would substantially reduce the size and the duration of clinical trials. This requires validation of surrogate markers showing that treatment induced change in the marker reliably predicts the magnitude of change in the clinical outcome. The surrogate marker must also reflect the biological mechanism for the effect of treatment on the clinical outcome. “Biological age” is a superficially attractive marker for such trials. However, it is essential to establish that treatment induced change in biological age reliably predict the magnitude of benefits in the clinical outcome. Reaching consensus on clinical outcomes for geroscience trials and then validating potential surrogate biomarkers requires time, effort, and coordination that will be worthwhile to develop surrogate outcomes that can be trusted to efficiently test the value of many anti-aging treatments under development.

## Introduction 

Geroscience is an emerging discipline seeking to evaluate the hypothesis that targeting aging biology can delay the onset of many age-related health conditions simultaneously thereby increasing health-span. Current intervention targets include a variety of aging hallmarks including senescent cells, mitochondrial function, mTOR, and stem cell depletion [1–4]. Ultimately, randomized clinical trials will be required to establish with rigor whether the targeting aging biology is effective and safe. By hypothesis, such interventions will simultaneously affect many diseases and health conditions, a situation that differs from traditional drug development approaches which target specific pathways related to specific diseases. In parallel, researchers are using a variety of statistical methods and biologic materials to identify biomarkers of aging [5–8]. An important goal for this research is to identify biomarkers that could be used to screen geroscience-based interventions. The availability of validated surrogate biomarkers would obviate the need conduct large, lengthy, and costly trials to establish treatment efficacy.

Trial design and biomarker discovery converge because putative biomarkers are evaluated by their ability to serve as surrogates for specific clinical endpoints. However, there is neither a consensus nor a process to generate consensus on what would constitute an acceptable endpoint for a geroscience-based prevention trial. Thus, current work to identify biomarkers is not aligned with the goal of accelerating the evaluation of promising interventions. We discuss possible clinical endpoints, the role that biomarkers might play in accelerating the pace of research in this area, and outline some critical steps to promote the alignment between these areas of research.

## Health outcomes

Table 1 presents alternative strategies for selecting endpoints for geroscience-based trials. Each is age-related, and it is reasonable to posit that stronger associations with age indicate that the endpoint is more likely to be responsive to interventions that slow aging. In selecting such an endpoint, several considerations should be born in mind.

**Table 1:** Potential “clinical” endpoints for clinical trials of treatments intended to slow aging

Health-related endpoint	Advantages	Disadvantages	Comment
Total mortality	Clinically important High face validity Aligns with experiment on lifespan model organisms	Rare: requires a large sample size and long duration Total mortality is comprised of diverse causes; an intervention might influence only a few causes of death	A collection of specific causes of death might be more specific to a mechanism of biological aging
Disability-free survival [19]	High salience to older adults	Difficult to operationalize Self-reported	Recovery is possible, so disability status may change over time
Specific diseases	A positive result can lead to an FDA-approved clinical indication	The treatment mechanism may not be related to the biology of aging	Reducing the risk of one disease may not reduce other aging-related conditions
Advancing multimorbidity [20, 21]	Increases event rates compared to single diseases More aligned with the geroscience hypothesis	Power driven by more common diseases Time to benefit may vary for different components	What morbidities should be included for geroscience-based trials is unclear
Frailty or deficit indices [21]	Large number of items increases event rate	No standardized tool available Not all items are of equal importance Poorly selected items will add variability or may have contrary relationship with the intervention	A more general version of advancing mulltimorbidity Directly operationalizes one view of aging as the accumulation of damage [22]
Frailty phenotype [23]	An important pre-disability state Large supporting literature	The 5-item version may be relatively insensitive to change [24]	

### Links to aging biology

Outcomes must be linked, in part, to fundamental biological processes of human aging. This information will typically come from model systems where the impact of perturbing aging biology can be rigorously evaluated. However, animals are poor models of many human diseases, and genetically manipulated models for one disease may not be relevant for other diseases. It may also be true that different underlying biologic mechanisms may relate to different sets of diseases/health conditions. For example, telomere length may be more important for cancer while mitochondrial function might be more important for heart failure. Human observational research can link putative aging biomarkers to patterns of human disease.

### Salience to different constituencies

Ideally, trial endpoints will be selected such that a successful trial would be persuasive to regulators, insurers, and broad section of the medical community. Each constituency may value different things. From a regulatory perspective, the US Food and Drug Administration requires that drugs being evaluated for potential approval must affect how a patient feels, functions, or survives. Reductions in health care expenditures would be salient to insurers and policy makers. The preservation of physical and cognitive function is highly valued by older adults.

### Feasibility

Complex, intensive, and expensive procedures, such as MRI or cardiopulmonary exercise testing, may be feasible for small trials. In general, meaningful health endpoints for geroscience trials would be ascertainable by interview, for example, about activities of daily living for disability, and by questions combined with simple tests, such as gait speed and change in body weight, for frailty. Incident diseases, such as cardiovascular disease are feasible to collect but may require validation in medical records.

The sample size and duration of trials is generally the most critical determinant of feasibility. Sample sizes for incident health events, such as a diagnosis of Alzheimer’s disease, are generally very large and require long follow-up because these age-related diseases are uncommon. Some diseases have a long prodromal period and the benefits of even a successful treatment might not be evident for a number of years. To increase the number of aging-related events, trials with incident clinical outcomes are conducted in participants who are older and have high risk of the outcome. The results, however, would generalize to a small fraction of the people who could benefit from such interventions changes earlier in life.

### Composite endpoints

Endpoints for trials of treatments that affect fundamental processes of aging may be designed to reduce the development of important health conditions that are not specific diseases, for example, disability, frailty, or even death. Endpoints that include a composite of multiple effects, such as multi-morbidity or accumulation of physical and cognitive deficits (deficit accumulation indices), have the advantages of higher rates requiring proportionately smaller sample sizes. If all, or the vast majority of, items reflect the effect of the treatment, then inclusion of all such endpoints can increase the frequency of the endpoint thereby reducing the sample size required to detect change in that index. On the other hand, a process such as cell senescence might influence only a subset of the items in the index. Sometimes, a treatment may have opposing effects on components of an index. In this case, a composite endpoint may reduce the sensitivity of the index and the size of the treatment effect. Caveats related to the use of composite endpoints in clinical trials are reviewed elsewhere [9].

## Biomarkers and surrogate markers

Biomarkers as endpoints of Geroscience trials are rightfully popular because they can serve several purposes in the development and testing of potential anti-aging interventions. There are several types of biomarkers, and a framework for identifying promising candidates for geroscience trials has been published [10–12].

### Response biomarkers

**Fig. 1:**
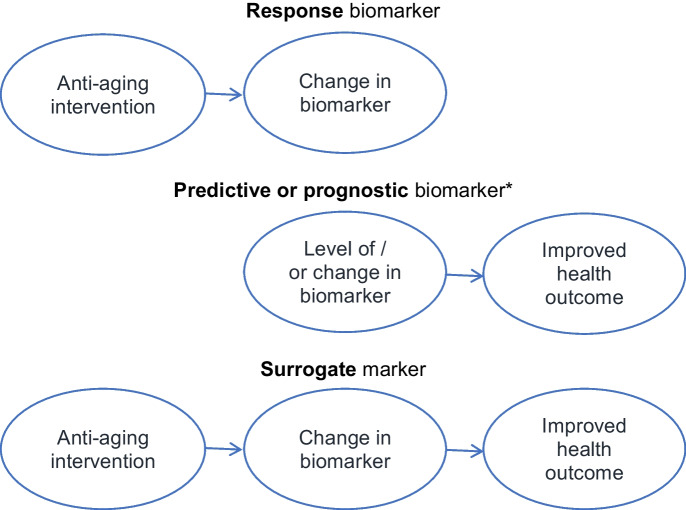
A marker that changes in response to a treatment is a *response biomarker*. A biomarker that predicts a health outcome is a predictive biomarker if it predicts the incidence of a health outcome and prognostic if it predicts the health outcome in patients who have the condition. A marker may be both a response and predictive biomarker but fail to be a valid surrogate marker, generally because the biomarker does not reflect a causal biological mechanism for the potential effect of the treatment on the heath outcome. A surrogate marker is valid if treatment changes the biomarker by the same mechanism by which the marker predicts and treatment improves the health outcome

Especially in the development of treatments, *response biomarkers,* such as senescence associate secretory phenotype (SASP) biomarkers, provide proof of concept that the treatment has a desired biological effect on target mechanisms of aging (killing senescent cells) or on their manifestations (e.g., 19). Establishing that a biomarker is a response biomarker establishes the first part of the pathway for a biomarker to be a potential surrogate (Fig. 1).

### Predictive and prognostic biomarkers

Predictive markers are an essential link in the pathway from treatment to change in a health condition (Fig. 1). Demonstrating that *predictive biomarkers*, such as quantity of Tau on brain scans, predicts the health outcome, such as incidence of the diagnosis of Alzheimer’s disease (AD), and provides support that the property measured by the marker may be a cause of the outcome.

However, a predictive marker is *not* necessarily a surrogate marker. Showing that change in the predictive marker is associated with change in the health endpoint strengthens the possibility but does not reliably show that the predictive marker is a true surrogate marker. What matters to establish that a biomarker is a surrogate is that *treatment induced change* in the biomarker predicts change in the health outcome. The same applies to *prognostic biomarkers*. This type of marker pertains to individuals who already have the health condition; the marker or change in that marker predicts change in the severity or level of the condition.

*Predictive and prognostic biomarkers* are useful in the design of trials by estimating a potential participant’s risk. This allows the inclusion of participants at high risk of the outcome. Ideally, a marker used to include participants is in the biological pathway from treatment effect to health outcome. Predictive markers may have clinical benefits by estimating a patient’s risk of the condition and, therefore, the magnitude of benefit from the treatment.

*Surrogate markers* are very attractive because trials with biomarker endpoints generally may require a much small number of participants, shorter follow-up times, and, therefore, much lower cost than trials with clinical or health outcomes (Table 1). Besides relatively low cost, such trials could afford to enroll a broader population at lower risk of a clinical outcome, thereby extending the generalizability of the results of a treatment trial to more people.

To be a valid surrogate, treatment-induced change in the marker must reliably predict a change health endpoint (Fig. 1). This requires that the biomarker accurately reflects the biological property that leads to the health effect. Thus, the biological effect of the treatment, the change in the marker, and change in the health outcome must all line up in the same causal pathway (Fig. 1).

### An example of a valid surrogate

**Fig. 2:**
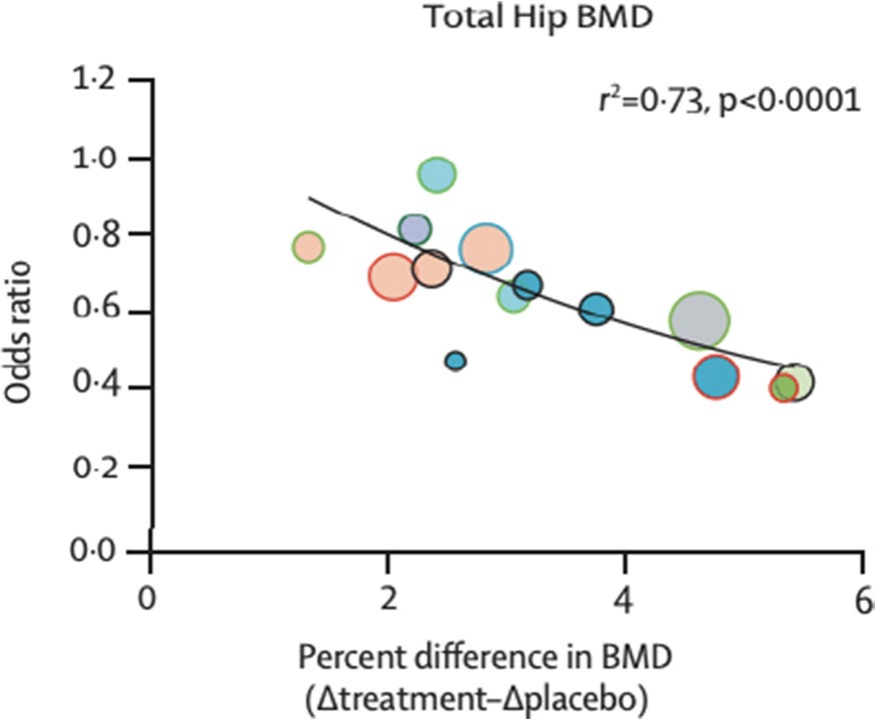
A meta-analysis of multiple trials of anti-resorptive treatments for osteoporosis demonstrated a strong (*R*^2^ = 0.73) correlation between absolute amount of increase in total hip bone density (BMD) and odds ratio of reduction in risk of hip fracture. This established change in BMD as a valid surrogate marker for the effect of treatment on risk of fracture

Bone density (BMD) of the hip is an example of a valid surrogate marker. Numerous clinical trials of the effect of treatment on change in hip BMD. Numerous very large (typically 5 to 10,000 women) randomized clinical trials with 3- to 5-year follow-up showed that treatments reduced the risk of hip and other types of fractures. This established two parts of the pathway but left uncertainly about whether treatment-induced improvement in BMD reliably predicts reduction in fracture risk. The large meta-analysis combining all clinical trials demonstrated a strong (*R*^2^ = 0.73) correlation between change in hip BMD and reduction in risk of hip fracture (Fig. 2a) connecting the parts of the causal pathway (Fig. 2b) and validating hip BMD as a surrogate marker. When hip BMD is officially accepted by the FDA, future trials of new treatments for osteoporosis will be much smaller and shorter—for example, a few hundred for 2 years—to quantify the effect of treatment on hip BMD to win FDA approval as a treatment to reduce hip fracture risk [13].

### Examples of failed surrogates

It is often assumed that showing that a biomarker, or change in that marker, predicts the health endpoint establishes that biomarker as a “surrogate.” The assumption is sometimes wrong and misleading. There are many examples of biomarkers that were trusted to be surrogate markers because treatment improved the marker and the marker had been shown to predict risk of the disease. For example, intensive hypoglycemic treatments, such as oral hypoglycemics and insulin, substantially reduce fasting glucose and HbA1c levels. Those levels predict the risk of cardiovascular disease. However, the belief that HbA1c was a surrogate marker was contradicted by findings from the ACCORD trial that diabetics randomly assigned to intensive treatment that reduced HbA1c levels in fact increased the risk of CVD events [14]. This result taught that intensive treatment must have other effects, besides change in HbA1c, that are not on the causal pathway, that resulted in the increased risk of CVD.

Similarly, a promising treatment for sarcopenia, MK-0773, substantially increased lean body and appendicular mass, but in a randomized clinical trial, the treatment had no effect on leg press strength, gait speed, or other health endpoints [15]. This suggests that change in lean mass does not increase muscle strength and function perhaps because “lean mass” is a poor measure of skeletal muscle mass.

The concentration of amyloid in the brain is associated with the risk and progression of dementia. Brain amyloid has been the target of numerous trials of treatments to prevent or reduce the progression of dementia. Consistently, trials that have found that reduction in brain amyloid have failed to have beneficial effects on cognitive function or risk of dementia [16].

## Implications for the use of “biological age” in clinical trials

The associated Conference on Biological Age reviewed several types of measuring biological aging [17]. Epigenetic age has become the most popular approach; derived from patterns of DNA methylation, commonly used epigenetic clocks are highly correlated with calendar age. Ideally, the difference between biological and calendar age, called “age acceleration,” predicted a variety of health outcomes. [e.g., 7] If an epigenetic clock—or other type of biological clock—has these properties, it is tempting to leap to the conclusion that the clock (or another type of biological age) is a “surrogate marker.” However, it is a very long jump from these findings to validation that biological age is a surrogate marker.

For biological age, or specifically, epigenetic age to be a valid surrogate, the treatment, epigenetic age, and the health outcome must be part of the same causal biological pathway (Fig. 1). If not, a treatment may alter “biological age” without affecting the causal mechanism that changes the endpoint (Fig. 1). In this case, using biological age as the endpoint of a trial is likely to produce misleading results about its effects on health outcomes.

Therefore, a key issue for the use of epigenetic age—or other measures of biological age—is understanding the mechanism for its association with calendar age and with health outcomes. Different epigenetic clocks may have different mechanisms [18]. If an epigenetic clock reflects multiple biological mechanisms, the overall clock is likely to be less sensitive to interventions that influence only one. Without an understanding of the biological mechanisms, it is uncertain and unlikely that biological age will be a valid surrogate marker for treatments that target a specific process of aging.

## The future

The use of biological age as an endpoint for clinical trials is just beginning. There are several practical but essential steps to validate that epigenetic age—or any type of biological age—is a valid surrogate marker.

There must be consensus about health outcomes to use in trials targeting mechanisms of aging, so results can be compared and pooled to provide power for analyses of potential surrogacy and test that the effect of treatment on a marker generalizes beyond more than one treatment. Until there is agreement about the best endpoint measurement trials should consider including several approaches as exploratory endpoints.

Clinical trials must be shown to have a beneficial effect on the health outcome. The trials must include measurement of change in potential surrogate outcomes. As the best biomarkers may yet to be discovered, it is important that trials store of blood, tissues, and images for future analysis correlating change in the marker with change in the health outcome.

There must be an infrastructure to support this important process. Ideally, a center would be designated to collect seek consensus on standard geroscience endpoints that would then be included in all geroscience trials. It is similarly important for trials to use similar and standardized measurements of biomarkers that could be maintained in a central resource. Critically, data from all trials must be made available for meta-analyses of changes in biomarkers and changes in health outcomes.

This process is slow but necessary to provide confidence that showing that a treatment that improves a biomarker will have the expected health benefits. There is an intense thirst for treatments to slow aging. A new treatment that slows a measurement of “biological age” that is *not* a true surrogate is likely to be widely used but ineffective or even harmful. Therefore, it is worth the effort and wait to establish “surrogate” markers whose changes can be trusted to translate into health benefits.
